# High-Dimensional Gene–Environment Interaction Analysis

**DOI:** 10.1146/annurev-statistics-112723-034315

**Published:** 2024-09-11

**Authors:** Mengyun Wu, Yingmeng Li, Shuangge Ma

**Affiliations:** 1 School of Statistics and Management, Shanghai University of Finance and Economics, Shanghai, China; 2 Department of Biostatistics, Yale School of Public Health, New Haven, Connecticut, USA

**Keywords:** gene–environment interaction, hypothesis testing, variable selection, dimension reduction, marginal and joint analysis

## Abstract

Beyond the main genetic and environmental effects, gene–environment (G–E) interactions have been demonstrated to significantly contribute to the development and progression of complex diseases. Published analyses of G–E interactions have primarily used a supervised framework to model both low-dimensional environmental factors and high-dimensional genetic factors in relation to disease outcomes. In this article, we aim to provide a selective review of methodological developments in G–E interaction analysis from a statistical perspective. The three main families of techniques are hypothesis testing, variable selection, and dimension reduction, which lead to three general frameworks: testing-based, estimation-based, and prediction-based. Linear- and nonlinear-effects analysis, fixed- and random-effects analysis, marginal and joint analysis, and Bayesian and frequentist analysis are reviewed to facilitate the conduct of interaction analysis in a wide range of situations with various assumptions and objectives. Statistical properties, computations, applications, and future directions are also discussed.

## 1. INTRODUCTION

Gene–environment (G–E) interactions have been recognized as significantly contributing to the etiology, progression, and biomarkers of complex diseases in addition to the main genetic and environmental effects (McAllister et al. 2017). A sample pivotal statement was provided by Alonso-Curbelo et al. (2021, p. 648) in *Nature*: “The initiation of cancer is facilitated by interactions between genetic and environmental insults.” There is much evidence to support this idea. For example, male mice exposed to Cr(III) chloride two weeks before mating exhibited a marked increase in the percentage of unmethylated copies of the 45S ribosomal RNA gene in their sperm. This epigenetic change was associated with an altered incidence of neoplastic and nonneoplastic alterations in the tissues of their progeny (Cheng et al. 2004). Moreover, experiments showed that when peripheral blood mononuclear cells were exposed to TLR7 ligands, female cells produced more interferon alpha than male cells. Such a heightened activity of the immune system could be a contributing factor to the increased vulnerability of women to autoimmune diseases compared with men, highlighting the complex interactions between gender and gene expression levels (Berghöfer et al. 2006).

In published studies, environmental factors are commonly classified into five categories. The first category encompasses objective environmental variables such as air quality and UV exposure. Another category includes demographic characteristics, such as age, gender, and weight. Additionally, personal habits, such as smoking and alcohol consumption, are often considered a significant category of factors. Another category includes social risk factors, such as financial insecurity and housing insecurity. Clinical variables may also serve as important environmental factors, such as the history of hypertension and high cholesterol. Genetic factors commonly encompass gene expression levels, single nucleotide polymorphisms (SNPs), and, in some cases, copy number variations and methylation, among others. Typically, methodologies for investigating G–E interactions are versatile, being applicable to multiple types of omics measurements. However, some studies specifically focus on the scenario where SNPs are densely clustered in very short chromosomal regions, which leads to modeling them as continuous sequence data and adopting functional data analysis approaches (Fan et al. 2013, Jiang et al. 2021, Chiu et al. 2022, Li et al. 2022, Wu et al. 2023a). These approaches are not suitable for other types of omics measurements.

In the literature, there are multiple reviews on genetic interactions that discuss this topic from various perspectives, where one or both of gene–gene interactions and G–E interactions have been examined. Among those focused on G–E interactions, a family of studies reviews the evidence that explains how environmental factors interact with the human genome in the cases of specific types of diseases, such as Manuck & McCaffery (2014) for psychological traits and disorders, Rudolph et al. (2016) for breast cancer, and Migliore & Coppedè (2022) for Alzheimer’s disease. Some other studies, such as those of Thomas (2010) and Han & Chatterjee (2018), concentrate on the study designs for G–E interaction analysis, including the case-only design, family-based association tests, two-phase case-control design, and countermatching. In addition, some studies are concerned with particular techniques. For example, Wu & Ma (2019a) examine robust G–E interaction analysis techniques, particularly addressing model misspecification and outliers/contamination in response variables and covariates. Zhou et al. (2021) investigate G–E studies from the viewpoint of variable selection with penalization techniques. Moreover, Miao et al. (2024) present statistical methodologies that mostly take advantage of testing techniques.

**Figure 1:**
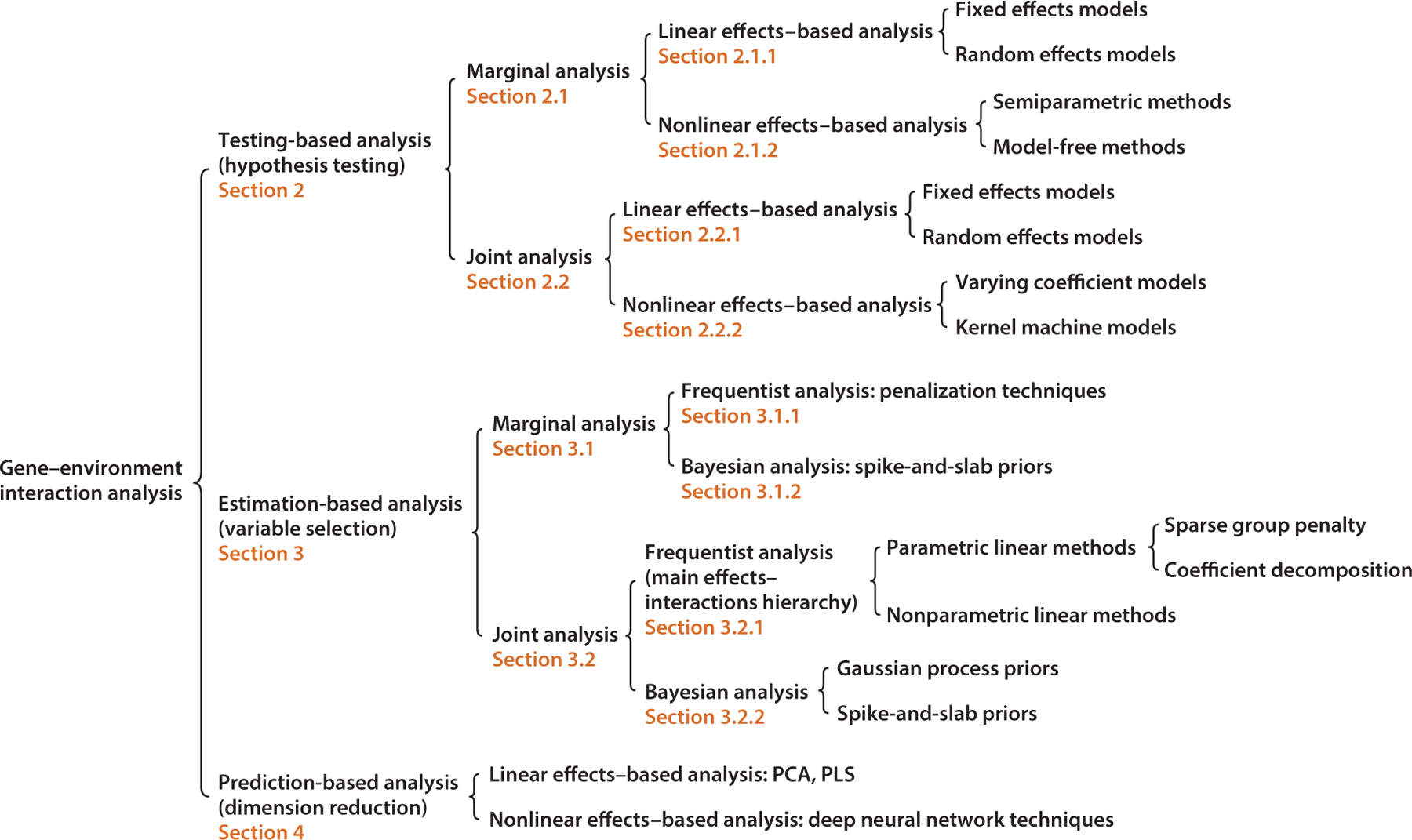
Overview of the methodologies discussed in this article for analyzing gene–environment interactions. Abbreviations: PCA, principal component analysis; PLS, partial least squares.

In this article, we take a different perspective and aim to provide a selective review that covers the existing statistical approaches for addressing the estimation and identification of G–E interactions, with particular attention to the high dimensionality of genetic factors. The methodological developments are classified into three generic analysis frameworks, namely testing-based, estimation-based, and prediction-based, with three main statistical techniques: hypothesis testing, variable selection, and dimension reduction. Specifically, testing-based methods involve hypothesis testing, followed by multiple-testing corrections, with the goal of testing whether interaction effects exist or not. Estimation-based methods utilize variable selection techniques to achieve sparse estimators, aiming to select interactions with none-zero effects and quantify their sizes. Different from these two frameworks, which can identify important interactions with sound interpretability, prediction-based methods conduct dimension reduction with the goal of achieving accurate prediction. Considering the notable differences across these frameworks, below we review each separately. For the testing-based and estimation-based frameworks, we further examine whether a small or a large number of genetic factors are analyzed at a time, which leads to marginal and joint analysis, respectively. In addition, both fixed and random effects as well as linear and nonlinear effects are investigated. For the estimation-based framework, we take a closer look at the underlying assumptions and examine both Bayesian and frequentist analysis. An overview of the methodologies is shown in Figure 1. Beyond the methodological developments, we also provide brief discussions on statistical properties, applications, computations, and future directions.

## 2. TESTING-BASED ANALYSIS

### 2.1. Marginal Analysis

In testing-based marginal analysis, as the number of variables (interaction and main effects) analyzed each time is considerably smaller than the sample size, the ordinary hypothesis testing is often adopted, and the important interactions are identified based on the testing results. In this section, we primarily review approaches for testing the interaction term or terms for each genetic factor. It should be noted that, although the dimensions of environmental factors are typically low here, genetic factors usually have high dimensions, necessitating multiple tests. Therefore, multiple comparisons generally need to be accounted for to control the overall error rate, and various methods have been used for adjustment. For example, the Benjamini–Hochberg procedure is used by Moore et al. (2019) and Ma et al. (2011b) to control the false discovery rate (FDR). Moreover, permutation testing methods are employed by Hahn et al. (2003) and Hou et al. (2019), while Bonferroni correction is utilized by Kerin & Marchini (2020).

#### 2.1.1. Linear effects–based analysis.

For the ith sample, denote $y_{i}$ as the response, $G_{ij}$ as the measurement of the jth genetic factor for j=1,…,p, and $E_{ik}$ as the measurement of the kth environmental factor for k=1,…,K. Most of the marginal analysis methods are based on the generalized linear regression model:

1. $$g\left(\mu_{i},j_{k}\right)=E_{ik}\alpha_{k}+G_{ij}\beta_{j}+\left(G_{ij}\times E_{ik}\right)\theta_{kj},$$

where $\mu_{i,jk}=E\left(y_{i}|E_{ik},G_{ij}\right)$ is the condition expectation of $y_{i}$ given $E_{ik}$ and $G_{ij}$; $g\left(\cdot \right)$ is a canonical link function; and $\alpha_{k}$, $\beta_{j}$, and $\theta_{kj}$ represent the main environmental, main genetic, and G–E interaction effects, respectively. The hypothesis test,

$$H_{0}:\theta_{kj}=0\;\text{and}\;H_{1}:\theta_{kj}\neq 0$$

is then conducted for each pair of $\left(j,k\right)$, followed by multiple-testing corrections. Various types of link functions and tests have been developed for accommodating different types of outcomes and study designs. A popular type of study focuses on the case-control design with a binary outcome $y_{i}=1$ for case and $y_{i}=0$ for control. In these studies, logistic regression with $g\left(\mu_{i,jk}\right)=\text{logit}\left(\mu_{i,jk}\right)=\log\left(\frac{\mu_{i,jk}}{1-\mu_{i,jk}}\right)$ is usually adopted together with likelihood ratio tests (LRTs) (Kraft et al. 2007, Thomas 2010, Han et al. 2012).

Despite considerable successes, these studies are not sufficiently powerful and robust due to their strong assumptions. Many efforts have been devoted to addressing these limitations for case-control studies. Examples include the work of de Rochemonteix et al. (2021), who incorporate the trend effect of a genotype and the G–E independence assumption into logistic regression and develop a retrospective LRT (LRT-R) to enhance power. Westerman et al. (2021) include a robust (sandwich) variance estimate in the calculation of score tests for marginal genetic effects and G–E interaction effects to achieve a more robust inference, referred to as GEM (which stands for gene–environment interaction analysis for millions of samples). Another example adopts a two-step strategy (Dai et al. 2012, Kawaguchi et al. 2023), which first filters out genetic variations that are not as important and then tests the most intriguing variants for G–E interactions to reduce the burden of multiple testing. Under this strategy, an important rationale is that a genetic factor having a G–E interaction effect should also have a main effect, which is known as the main effects–interactions hierarchy.

In addition to binary outcome, continuous phenotype has also been commonly investigated. For example, Majumdar et al. (2020) consider three lipids, low-density lipoprotein, high-density lipoprotein, and triglycerides, in the UK Biobank as the continuous phenotypes and examine the interactions between the frequency of alcohol consumption and SNPs. A two-step approach similar to that of Dai et al. (2012) and Kawaguchi et al. (2023) is developed, which introduces two multivariate linear regressions to model the main genetic effects of SNPs and both the main effects and the interactions between SNPs and environmental factors in the first and second steps, respectively, and then combines the *p*-values obtained from the two steps to identify the SNPs with important interaction effects.

The aforementioned approaches are mostly based on fixed-effects models, which are limited by an inability to handle sample relatedness, leading to ineffective model estimation and interaction identification. To alleviate this problem, a family of approaches based on linear mixed models (LMMs) have been developed. Denote by y, G, and E the n×1 vectors consisting of $y_{i}\text{s}$, $G_{i}\text{s}$, and $E_{i}\text{s}$ of n samples. Here, we omit the dependence on $\left(k,j\right)$ for the *k*th environmental and jth genetic factors to simplify notation. The LMM for G–E interaction analysis usually has the following formulation:

2. $$\text{y}=G\beta +\left(G⊙E\right)\theta +g+\epsilon ,$$

where ⊙ denotes the element-wise product, θ is the fixed G–E interaction effect, $g∼N\left(0,K\sigma_{g}^{2}\right)$ is an n×1 vector of the random effects consisting of main genetic and G–E interaction effects with the matrix K accounting for genome-wide variants genetic effects and sample relatedness, and $\epsilon ∼N\left(0,I_{n}\sigma_{\epsilon }\right)$ is an n×1 vector of residuals with ${\text{I}}_{n}$ being an n×n identity matrix. Under the LMM, multiple types of testing have been developed for identifying important G–E interactions, including the variance component test [StructLMM, which stands for structured linear mixed model (Moore et al. 2019)], the robust F test [LEMMA, which stands for linear environment mixed model analysis (Kerin & Marchini 2020)], and the Wald test with a sandwich correction (fastGWA-GE; Zhong et al. 2023).

Compared with binary and continuous outcomes, there are fewer G–E interaction studies tailored to survival response, which may be more challenging due to the characteristics of survival data, such as nonnegative distributions and censoring. Relevant methodological developments include the work of Xu et al. (2019), who introduce a censored quantile partial correlation (CQPCorr) to measure the importance of interactions while properly controlling for the main genetic and environmental effects, followed by a permutation-based test. Additionally, Wang & Yang (2022) introduce a nonparametric inverse probability-of-censoring weighted Kendall’s partial correlation approach (IPCW-pcorr), which enjoys robustness against model misspecification and outliers.

#### 2.1.2. Nonlinear effects–based analysis.

The linearity assumption can be violated due to the complex biological mechanisms that can induce nonlinear G–E interactions. A series of semiparametric model-based tests have been developed, which mostly focus on modeling complex nonlinear effects of continuous environmental exposures to indirectly explore nonlinear G–E interactions (Maity et al. 2009). Among them, the varying coefficient (VC) model is perhaps the most popular, which is specified as

3. $$y_{i}=\alpha_{k}\left(E_{ik}\right)+G_{ij}\beta_{j}+\theta_{kj}\left(E_{ik}\right)G_{ij}+\epsilon_{i},$$

where $\alpha_{k}\left(E_{ik}\right)$ and $\theta_{kj}\left(E_{ik}\right)$ are two smooth nonlinear functions. Under the model in Equation 3, the spline approximation technique is often adopted for estimation, and the testing of functional coefficient $\theta_{kj}\left(\cdot \right)=0$ can then be transferred into a traditional parametric test on the coefficients of the spline basis functions, such as wild bootstrap–based testing (Ma et al. 2011b) and the LRT (Zhou et al. 2023b). Liu et al. (2020) extend the model in Equation 3 and develop a partially linear VC model to accommodate both discrete and continuous environmental factors with parametric and nonparametric components. They propose a generalized LRT to jointly test the two components, making this technique more advanced than the aforementioned ones because it simultaneously assesses the effects of linear and nonlinear G–E interactions.

In addition to semiparametric modeling, model-free approaches have also gained considerable attention in recent years and are becoming popular. These approaches make no assumptions about the relationship between the phenotypes and genetic and environmental factors. A representative approach is multifactor dimensionality reduction (MDR) (Hahn et al. 2003), which was first developed for case-control studies. In this approach, for each pair of environmental and genetic factors, the possible multifactor classes are labeled as either high-risk or low-risk based on the ratio of the number of cases to the number of controls. The ability of the new one-dimensional G–E interaction variable to classify and predict disease status is then assessed using cross-validation and permutation testing. Extensions of MDR have been widely examined in the literature, such as the generalized linear model– (GLM-)based MDR for dichotomous and continuous responses (Lou et al. 2007) and the proportional odds model–based MDR for ordinal phenotypes (GMDR; Hou et al. 2019). The other type of model-free G–E interaction analysis approach is mostly based on the information-theoretic technique. For example, Wu et al. (2009) utilize mutual information that measures the dependence between two random variables and define the information measure of the interaction between a gene and an environmental variable as

4. $$I_{GE}=\sum_{i=0}^{2}\sum_{j=0}^{1}P\left(G=i,E=j|D=1\right)\log\frac{P\left(D=1|G=i,E=j\right)/P_{D}}{P\left(D=1|G=i\right)\text{/}P_{D}P\left(D=1|E=j\right)/P_{D}},$$

where the discrete genetic factor G has three genotypes coded as 0, 1, and 2; the environmental exposure is coded as E=1 (if exposed) and E=0 (if otherwise); D is an indicator of disease; and $P_{D}=P\left(D=1\right)$. A test statistic is then developed to test whether $I_{GE}=0$, which asymptotically has a $\chi_{2}^{2}$ distribution under the null hypothesis. Multiple follow-up studies, such as those of Fan et al. (2011) and Knights et al. (2013), extend Wu et al. (2009) and develop the entropy-based information gain approaches to analyze the interactions between multiple genetic and environmental factors.

### 2.2. Joint Analysis

In joint analysis, multiple genetic factors are collectively considered. This can partially address the limitation of the single-marker G–E interaction test that does not interrogate the joint effects of multiple genetic factors. Under this framework, most approaches are model-based and can be classified into linear effects–based and nonlinear effects–based.

#### 2.2.1. Linear effects–based analysis.

As in marginal analysis, the GLM is the most commonly used technique in joint analysis. Denote as $G_{i}={\left(G_{i1},\ldots ,G_{ip}\right)}^{⊤}$ a vector of p genetic markers and ${\text{S}}_{i}={\left(G_{i1}E_{i},\ldots ,G_{ip}E_{i}\right)}^{⊤}$ a vector of G–E interactions for the ith sample (we omit the dependence on k for the kth environmental factor to simplify notation). Consider the marker set and environment interaction GLM:

5. $$g\left(\mu_{i}\right)=E_{i}\alpha +G_{i}^{⊤}\beta +S_{i}^{⊤}\theta ,$$

with the testing $H_{0}:\text{θ}=0$, where $\mu_{i}=E\left(y_{i}|E_{i},G_{i}\right)$, and $\theta ={\left(\theta_{1},\ldots ,\theta_{p}\right)}^{⊤}$ is a coefficient vector for the interaction effects. Kim et al. (2019) examine the three joint tests using the Wald, likelihood ratio, and score statistics based on Equation 5. It has been demonstrated by Kim et al. (2019) that, in the joint test for multiple interactions, particularly for a binary trait, the selection of statistic is important. With an increase in interaction parameters in logistic models, the Wald test and LRT statistics show deflation and inflation, respectively, but the score statistic remains consistently more robust.

In contrast to the studies focused on fixed effects, Lin et al. (2013) derive an equivalent testing $H_{0}:\tau^{2}=0$ by assuming $\theta_{j}\text{s}$ are independent and identically following an arbitrary distribution with mean zero and common variance $\tau^{2}$ to accommodate random effects, leading to a generalized linear mixed model (GLMM). A variance component test using a score test is then introduced. Under a similar GLMM framework, Yang et al. (2019) introduce an adaptive sum of powered score tests, referred to as adaptive gene–environment interaction (aGE) test, which can control the type I error rate in the presence of a large number of neutral variants. Wang et al. (2017) take a further step and propose a set-based mixed effect model, referred to as mixed effect model for gene–environment interaction (MixGE), to incorporate both the fixed and random effects of G–E interactions, investigating homogeneous and heterogeneous contributions of sets of genetic variants and their G–E interactions. A score statistic is developed for simultaneously testing the terms associated with fixed and random effects.

Instead of jointly testing the effects of multiple G–E interactions, some studies conduct a single univariate test on an aggregated statistic as an enriched signal corresponding to a marker set. An example is that of Lu et al. (2014), who first compute the first principal component score on each candidate set of genetic factors as its corresponding aggregated statistic and then fit a linear regression model with the environmental factor, the aggregated statistic, and their interaction, followed by a *t*-test to examine the significance of the interaction term. Under a similar strategy, Hecker et al. (2022) derive robust interaction testing using sample splitting (RITSS), which employs an interaction score comprising the (weighted) sum of individual genetic variant/environmental factor product pairs, and utilizes a sample splitting strategy and a test statistic that are robust against misspecification of the main effects.

In joint analysis, the main effects–interactions hierarchy has been generally respected to improve estimation and interpretation. To effectively accommodate this hierarchy, for survival outcome, Liang et al. (2024) develop a hierarchical FDR control method based on the accelerated failure time model, where a weighted least squares plus debiased lasso approach is adopted for estimation and selection. This method is the first to carry out hypothesis testing across all high-dimensional main effects, followed by testing the interactions whose associated main effects have been rejected. By defining ${\text{FDR}}_{j}$ as the proportion of incorrectly rejected hypotheses for interaction effects conditional upon the rejection of the main effect hypothesis for the jth high-dimensional main effect, Liang et al. (2024) establish $\text{FDR}=\sum_{j=0}^{p}{\text{FDR}}_{j}$ as the overall FDR to be controlled, where ${\text{FDR}}_{0}$ represents the FDR for the main effects.

#### 2.2.2. Nonlinear effects–based analysis.

Nonlinear G–E interaction effects have also been investigated in joint testing–based studies, which are more challenging. As such, the relevant methodological developments are still limited. Commonly adopted techniques include the VC and kernel machine models. Specifically, as an extension of the marginal VC model (Equation 3) (Ma et al. 2011b), Sa et al. (2016) develop a VC principal component regression model:

6. $$y_{i}=\alpha \left(E_{i}\right)+\sum_{t=1}^{T}\theta_{t}\left(E_{i}\right)U_{it}+\epsilon_{i},$$

where $U_{i1},\ldots ,U_{iT}$ are the first T (sparse) principal components of the p genetic factors ${\text{G}}_{i}$. Then, testing G–E interactions can be formulated as $H_{0}:\theta_{1}\left(\cdot \right)=\cdots =\theta_{T}\left(\cdot \right)=0$ based on the model in Equation 6. Through a nonparametric technique, $\theta_{t}\left(\cdot \right)\text{s}$ are approximated by $\theta_{t}\left(E_{i}\right)=\sum_{l=1}^{L}\eta_{tl}B_{tl}\left(E_{i}\right)$, with ${\left\{B_{tl}\left(\cdot \right)\right\}}_{l=1}^{L}$ being the basis functions, and the testing problem is transferred into $H_{0}:\eta_{tl}=0$ for all t, l. A least-squares technique and LRT are further adopted for estimation and inference. Instead of focusing on the nonlinear effects of continuous environmental factors, Marceau et al. (2015) develop a kernel machine score test, FastKM, based on multi-kernel analysis to accommodate the nonlinear effects of genetic factors. Specifically, consider the matrix formulation of a GLM,

7. $$g\left(\mu \right)=E^{⊤}\alpha +\beta \left(G\right)+\theta \left(G,E\right),$$

where $\mu ={\left(\mu_{1},\ldots ,\mu_{n}\right)}^{⊤}$, $\beta \left(G\right)={\left(\beta \left(G_{1}\right),\ldots ,\beta \left(G_{n}\right)\right)}^{⊤}$, and $\theta \left(G,E\right)=\left(\theta \left(G_{1},E_{1}\right),\ldots ,\theta {\left(G_{n},E_{n}\right)}^{⊤}\right)$, with $\beta \left(\cdot \right)$ and $\theta \left(\cdot \right)$ being two nonparametric smooth functions representing the main effects of genetic markers and G–E interaction effects. By the representer theorem, $\beta \left(\cdot \right)$ and $\theta \left(\cdot \right)$ can be rewritten in dual form expressions as $\beta \left(\cdot \right)={\text{K}}_{\text{G}}\gamma_{\text{G}}$ and $\theta \left(\cdot \right)={\text{K}}_{\text{GEI}}\gamma_{\text{GEI}}$, where ${\text{K}}_{\text{G}}$ and ${\text{K}}_{\text{GEI}}$ are two n×n kernel matrices and $\gamma_{G}$ and $\gamma_{\text{GEI}}$ are n vectors of unknown parameters. An identity by state kernel is adopted for $K_{G}$, while $\theta \left(\cdot \right)$ is treated as random effects with $\theta \left(\cdot \right)∼N\left(0,\tau K_{\text{GEI}}\right)$. Using this representation, testing $H_{0}:\theta \left(\cdot \right)=0$ is equivalent to testing the null hypothesis $H_{0}:\tau =0$ via a variance component score test. As an “upgrade”, Zhao et al. (2019) introduces a composite kernel, which is constructed as a weighted average of two individual kernels corresponding to the genetic main effects and G–E interaction effects, for the overall genetic effects. The weights can be estimated data-dependently to effectively improve statistical power of the proposed restricted LRT.

## 3. ESTIMATION-BASED ANALYSIS

Similar to testing-based analysis, estimation-based analysis is based on the sparsity assumption and motivated by the fact that many or most genetic factors and G–E interactions are noise, and they do not contribute to the response and have zero effects. Regularization techniques have been commonly adopted for sparse estimation, where the identification of important G–E interactions is regarded as a variable selection problem. The popular methods can be generally classified as frequentist and Bayesian analysis.

### 3.1. Marginal Analysis

Unlike testing-based analysis, where the majority of the approaches are in the marginal analysis paradigm, most estimation-based analysis takes the form of joint analysis. The limited marginal analysis studies mostly examine linear effects under GLMs for multiple environmental factors $E_{i1},\ldots ,E_{iK}$ and one genetic factor $G_{ij}$:

8. $$g\left(\mu_{i,j}\right)=\sum_{k=1}^{K}E_{ik}\alpha_{k}+G_{ij}\beta_{j}+\sum_{k=1}^{K}G_{ij}E_{ik}\theta_{kj}=E_{i}^{⊤}\alpha +W_{ij}^{⊤}b_{j},$$

where $\mu_{i,j}=E\left(y_{i}|E_{i},G_{ij}\right)$, $W_{ij}={\left(G_{ij},E_{i1}G_{ij},\ldots ,E_{iK}G_{ij}\right)}^{⊤}$, and $b_{j}={\left(\beta_{j},\theta_{1j},\ldots ,\theta_{Kj}\right)}^{⊤}$.

#### 3.1.1. Frequentist analysis.

Under the model in Equation 8, frequentist analysis usually conducts estimation and selection by minimizing a penalized objective function consisting of two terms: loss function $\ell \left(\alpha ,b_{j}\right)$ and penalty function $\rho \left({\text{b}}_{j};\lambda ,\zeta \right)$ with a tuning parameter λ>0 and regularization parameter λ>0. Here, the penalty is imposed on ${\text{b}}_{j}$ and consists of the main genetic effect and G–E interactions, making $\beta_{j}$ and $\theta_{kj}\text{s}$ shrink to zero for some j. The jth genetic factor with a nonzero value of $\theta_{kj}$ is regarded as having an interaction with the kth environmental factor. In typical G–E interaction studies, the environmental factors are preselected and have a low dimension, and hence selection is not conducted on the environmental variables.

Multiple combinations of loss functions and penalty functions have been investigated. For example, Shi et al. (2014) introduce a rank-based loss function,

9. $$\ell \left(\alpha ,b_{j}\right)=-\frac{1}{n\left(n-1\right)}\sum_{i\neq l}I\left(y_{i}\geq y_{l}\right)I\left(E_{i}^{⊤}\alpha +W_{ij}^{⊤}b_{j}\geq E_{l}^{⊤}\alpha +W_{lj}^{⊤}b_{j}\right),$$

for a continuous outcome, which is not sensitive to model specification, and a penalty term $\rho_{\text{MCP}}\left(\left|\beta_{j}\right|;\lambda ,\zeta \right)+\sum_{k=1}^{K}\rho_{\text{MCP}}\left(\left|\theta_{kj}\right|;\lambda ,\zeta \right)$ with the minimum concave penalty (MCP)$\rho_{\text{MCP}}\left(t;\lambda ,\zeta \right)=\lambda \int_{0}^{\left|t\right|}{\left(1-\frac{x}{\lambda \zeta }\right)}_{+}\text{d}x$. The rank-based losses for binary outcome and censoring survival outcome have also been examined by Shi et al. (2014). In addition, Chai et al. (2017) propose a robust approach based on an exponential squared loss to accommodate data contamination or a mixture of distributions.

A few methods have been developed to accommodate the main effects–interactions hierarchy and achieve more interpretable estimation. For example, Zhang et al. (2020) consider the negative log-likelihood function for $\ell \left(\alpha ,b_{j}\right)$ and develop the sparse group MCP:

10. $$\rho_{\text{sgMCP}}\left(b_{j};\lambda ,\zeta \right)=\rho_{\text{MCP}}\left(∥b_{j}{∥}_{2};\sqrt{K+1}\lambda ,\zeta \right)+\sum_{k=2}^{K+1}\rho_{\text{MCP}}\left(\left|b_{kj}\right|;\lambda ,\zeta \right).$$

Here, the penalty term includes two parts, which are imposed on the $L_{2}$-norm of ${\text{b}}_{j}$ and absolute values of $b_{2j},\ldots ,b_{Kj}$, respectively. This automatically ensures that if $b_{kj}\neq 0$ for any k≥2 (that is, an interaction term), then $b_{1j}\neq 0$ (that is, the main genetic effect), respecting the main effects–interactions hierarchy. Ren et al. (2022) further propose a robust extension of Zhang et al. (2020) and adopt a γ-divergence loss function to accommodate contaminated data without assumptions on contamination distribution and proportion. In the aforementioned studies, MCP is adopted for building penalties for regularized estimation and variable selection. Other penalties can also be used, including, for example, the lasso family, bridge, and SCAD.

#### 3.1.2. Bayesian analysis.

Besides the regression-based frequentist models, a few marginal Bayesian models have been developed for identifying G–E interactions, with the advantage of conveniently providing uncertainty quantification based on the posterior samples from Markov chain Monte Carlo (MCMC). For example, consider the model in Equation 8 for continuous outcome with $g\left(\mu_{i,j}\right)=\mu_{i,j}$. Lu et al. (2021) assume a Laplace distribution for the random error and propose a marginal Bayesian least absolute deviation regression with the likelihood function as

11. $$f\left(y|\alpha ,\beta_{j},\theta_{j}\right)=\prod_{i=1}^{n}\frac{\tau }{2}\exp\left(-\tau \left|y_{i}-\sum_{k=1}E_{ik}\alpha_{k}-G_{ij}\beta_{j}-\sum_{k=1}^{K}G_{ij}E_{ik}\theta_{kj}\right|\right).$$

The spike-and-slab priors are introduced for Equation 11 with $\beta_{j}|s_{1}$, $\pi_{1}∼\left(1-\pi_{1}\right)N\left(0,s_{1}\right)+\pi_{1}\delta_{0}\left(\beta_{j}\right)$ and $\theta_{kj}|s_{2k}$, $\pi_{2}∼\left(1-\pi_{2}\right)N\left(0,s_{2k}\right)+\pi_{2}\delta_{0}\left(\theta_{kj}\right)$, where $\delta_{0}\left(\beta_{j}\right)$ and $\delta_{0}\left(\theta_{kj}\right)$ denote the spike at 0, leading to sparse estimation. The conjugate priors are assigned on the remaining parameters, facilitating the usage of Gibbs sampling for posterior inference.

### 3.2. Joint Analysis

Significant progress has also been made toward modeling all genetic factors and their interactions in one single model:

12. $$g\left(\mu_{i}\right)=\sum_{k=1}E_{ik}\alpha_{k}+\sum_{j=1}^{p}\left(G_{ij}\beta_{j}+\sum_{k=1}^{K}G_{ij}E_{ik}\theta_{kj}\right)=E_{i}^{⊤}\alpha +\sum_{j=1}^{p}W_{ij}^{⊤}b_{j}.$$

#### 3.2.1. Frequentist analysis.

Based on Equation 12, the loss function plus penalty function strategy has also been adopted for regularized estimation and variable selection. Compared with marginal analysis, joint analysis is usually more challenging due to the high dimensionality of the genetic factors and the need to respect the main effects–interactions hierarchy.

One popular strategy is utilizing the sparse group penalty, similar to Equation 10. A representative work (Liu et al. 2013) considers the negative log-likelihood function and sparse group MCP (sgMCP), defined as

13. $$\sum_{j=1}^{p}\rho_{\text{sgMCP}}\left(b_{j};\lambda ,\zeta \right)=\sum_{j=1}^{p}\left(\rho_{\text{MCP}}\left(∥b_{j}{∥}_{2};\sqrt{K+1}\lambda ,\zeta \right)+\sum_{k=2}^{K+1}\rho_{\text{MCP}}\left(\left|b_{kj}\right|;\lambda ,\zeta \right)\right),$$

to jointly accommodate *p* genetic factors. As discussed above, this penalty can effectively respect the main effects–interactions hierarchy. A number of extensions have been examined in the literature. For example, Wu et al. (2017) develop a nonparametric kernel-based data augmentation approach to address the missingness in environmental measurements in the model in Equation 12, leading to more accurate and more biologically meaningful findings. In addition, Wu et al. (2018) introduce LAD-hier (where LAD stands for least absolute deviation), which uses the least absolute deviation loss function with the sparse group lasso penalty and enjoys robustness properties against data contamination and outliers. In some other studies, additional information is incorporated to assist in more effective interaction analysis. Examples include the work of Wang et al. (2019) (psgMCP), who incorporate the existing literature information, and Fang et al. (2023), who incorporate pathological imaging data. Some effort has also been devoted to exploring the sparse group penalty. For example, Zemlianskaia et al. (2022) develop the gesso model, which uses penalty $\sum_{j=1}^{p}\left(\lambda_{1}∥b_{j}{∥}_{\infty }+\sum_{k=2}^{K+1}\lambda_{2}\left|b_{kj}\right|\right)$ with the $L_{\infty }$ group norm replacing the $L_{2}$ group norm in Equation 13 and new screening rules that eliminate a large number of variables beforehand, making joint G–E analysis feasible at a genome-wide scale.

Another popular strategy is to conduct a coefficient decomposition with $\theta_{kj}=\beta_{j}\gamma_{kj}$, incorporated with a sparse penalty imposed on $\left|\beta_{j}\right|$ and $\left|\gamma_{kj}\right|$ separately. As a result, if an interaction term is selected $\left(\beta_{j}\gamma_{kj}\neq 0\right)$, the corresponding main genetic effect must also be selected $\left(\beta_{j}\neq 0\right)$. Utilizing this strategy, Xu et al. (2018) develop a robust G–E identification approach using the trimmed regression technique, which has robustness against outliers and contamination in both response and predictors. Recently, the structure of genetic factors has attracted much attention, such as the adjacency structure of SNPs attributable to their physical adjacency on the chromosomes and the group or network structure of gene expressions attributable to their coordinated biological functions and correlated measurements. Taking advantage of the decomposition strategy, besides the sparse penalty, Pashova et al. (2017) introduce an additional pairwise fused lasso penalty $\sum_{j=1}\sum_{l\neq j}\left|\gamma_{kj}-\gamma_{kl}\right|$ to encourage the formation of groups of interactions. In addition, Wu et al. (2020) take a further step and consider various types of underlying structures of genetic factors in the analysis of both main effects and G–E interactions. Specifically, the spline-type penalties $\sum_{j=2}^{p-1}{\left[\left(\beta_{j+1}-\beta_{j}\right)-\left(\beta_{j}-\beta_{j-1}\right)\right]}^{2}$ and $\sum_{j=2}^{p-1}{\left[\left(\gamma_{k,j+1}-\gamma_{k,j}\right)-\left(\gamma_{k,j}-\gamma_{k,j-1}\right)\right]}^{2}$ are developed to accommodate the adjacency structure of SNPs, and the Laplacian-type penalties $\sum_{j∼l}a_{jl}{\left(\frac{\beta_{j}}{\sqrt{d_{j}}}-\frac{\beta_{l}}{\sqrt{d_{l}}}\right)}^{2}$ and $\sum_{j∼l}a_{jl}{\left(\frac{\gamma_{kj}}{\sqrt{d_{j}}}-\frac{\gamma_{kl}}{\sqrt{d_{l}}}\right)}^{2}$ are developed to accommodate the network structure of genes, where j~l denotes the connections in the network with $a_{jl}$ being the connection strength and $d_{j}=\sum_{l∼j}a_{jl}$, promoting the adjacent SNPs or connected genes to have similar main effects (interactions) associated with the response. In previous studies, only the interactions between one type of molecular measurement and environmental risk factor were analyzed, which may not be sufficient to describe complex biological mechanisms. To accommodate multiple types of molecular measurements, Xu et al. (2022) extend the decomposition strategy to multidimensional molecular measurements–environment interaction analysis to sufficiently account for their overlapping as well as independent information.

Besides the parametric linear methods, some nonparametric techniques have also been developed for accommodating nonlinear G–E interactions. As in the testing-based analysis, researchers are mostly interested in the nonlinear interaction effects with continuous environmental factors. For example, Wu et al. (2014) consider the partially linear VC model with the basis expansion approximation for smooth nonlinear function $\theta_{kj}\left(E_{ik}\right)$, which can be regarded as a joint version of the model of Liu et al. (2020). As opposed to utilizing the generalized LRT like Liu et al. (2020), Wu et al. (2014) adopt estimation-based analysis and introduce a group penalty for the group of spline coefficients corresponding to $\theta_{kj}\left(E_{ik}\right)$, achieving the goal of identifying important G–E interactions with nonzero $\theta_{kj}\left(\cdot \right)$. A robust extension of this work is further developed by Wu et al. (2015), who use a rank-based loss function to accommodate data contamination. With the consideration of reducing computational cost, the main effects–interactions hierarchy, which demands more complicated model formulations or penalties, is not accommodated by Wu et al. (2014, 2015). To address this limitation, under a similar partially linear VC model, instead of penalization, Wu & Ma (2019b) take advantage of the sparse boosting algorithm and design an updating strategy that only searches over those interactions with corresponding main effects already selected in the model, to respect the main effects–interactions hierarchy. The nonlinear effects of genetic factors have also been examined in recent published studies. Bhatnagar et al. (2023) develop a smoothing method for the genetic factors $G_{ij}\text{s}$, called the sparse additive interaction learning model (sail), via a projection onto a set of basis functions:

14. $$h_{j}\left(G_{ij}\right)=\sum_{l=1}^{m_{j}}\psi_{jl}\left(G_{ij}\right)\eta_{jl},$$

where $\psi_{jl}\left(\cdot \right)\text{s}$ are the basis functions and $\eta_{jl}\text{s}$ are the corresponding coefficients, respectively. Denote ${\text{ψ}}_{j}\left(G_{ij}\right)=\left(\psi_{j1}\left(G_{ij}\right),\ldots ,\psi_{j,m_{j}}\left(G_{ij}\right)\right)$, and then an additive interaction regression model is proposed with the form:

$$y_{i}=E_{i}^{⊤}\alpha +\sum_{j=1}^{p}\psi_{j}{\left(G_{ij}\right)}^{⊤}\beta_{j}+\sum_{j=1}^{p}\sum_{i=1}^{K}E_{ik}\psi_{j}{\left(G_{ij}\right)}^{⊤}\theta_{k,j}+\epsilon ,$$

where the main genetic effects and interactions are described by the vectors ${\text{β}}_{j}$ and ${\text{θ}}_{k,j}$, respectively. A decomposition strategy is adopted with $\theta_{k,j}=\beta_{j}⊙\gamma_{k,j}$, together with penalty $\lambda_{1}\sum_{j=1}^{p}∥\beta_{j}{∥}_{2}+\lambda_{2}\sum_{k=1}^{K}\sum_{j=1}^{p}∥\gamma_{kj}{∥}_{2}$, to accommodate the main effects–interactions hierarchy.

#### 3.2.2. Bayesian analysis.

There are a few Bayesian joint G–E interaction analysis studies, which are mostly concerned with nonlinear effects and data contamination and outliers. Some of them utilize Gaussian process priors to identify G–E interactions, such as the nonparametric Bayesian approach of Zou et al. (2010) for mapping multiple quantitative trait loci (QTLs). This method captures both genetic and nongenetic influences through an unspecified function, facilitated by a Gaussian process prior. It evaluates the significance of each QTL and environmental factor without explicitly modeling their interactions or main effects, providing a comprehensive analysis of its impact.

Instead of the conventional likelihood, Lobach et al. (2011) employ the pseudolikelihood function for case-control studies involving measurement errors in environmental variables and missing data in genetic variables. Since a direct application of traditional Bayesian techniques is not feasible due to the utilization of the pseudolikelihood function, Lobach et al. (2011) adopt both MCMC and a simple computational approach based on an asymptotic posterior distribution.

More recent studies usually exploit the spike-and-slab priors for identifying important G–E interactions. Examples include Bayesian spike-and-slab variable selection with structural identification (BSSVC-SI), proposed by Ren et al. (2020), which considers the partially linear VC model for investigating the nonlinear effects of continuous environmental factors and their interactions. After the basis expansion for the VC functions, a spike-and-slab Laplace prior is imposed on the groups of basis coefficients corresponding to the main genetic effects and interactions. However, this method may be challenged by heavy-tailed errors and outliers in the response variable. To address this limitation, Ren et al. (2023) develop a robust Bayesian sparse group selection with spike-and-slab priors (RBSG-SS), which employs a Bayesian formulation of the least absolute deviation regression and spike-and-slab priors for robust sparse group selection at both the individual and group levels. In addition, the issue of heavy-tailed distributions and outliers is addressed by Zhou et al. (2023a) using a Bayesian regularized quantile varying coefficient model with spike-and-slab priors (BQRVCSS), which accommodates nonlinear interactions between the effect modifiers and predictors. The method proposed by Ren et al. (2023) represents a special case of the Bayesian penalized quantile regression at the 50% quantile level, making the study of Zhou et al. (2023a) an extension of the approach proposed by Ren et al. (2023).

There are also a few Bayesian methods that can accommodate the main effects–interactions hierarchy, including the Bayesian hierarchical mixture model proposed by Liu et al. (2015), which simultaneously addresses gene–gene and G–E interactions. This method efficiently integrates the inherent hierarchical structure between main and interaction effects into a mixture model, effectively eliminating irrelevant interaction effects and resulting in more robust and streamlined models.

## 4. PREDICTION-BASED ANALYSIS

Dimension reduction is commonly employed for prediction-based analysis. It is valuable in scenarios where the primary objective is to enhance model prediction accuracy rather than explicitly identify specific G–E effects. Various dimension reduction techniques have been developed and can be classified as linear effects– and nonlinear effects–based analysis.

### 4.1. Linear Effects–Based Analysis

The linear effects–based analysis usually adopts classical dimension reduction techniques, such as principal component analysis (PCA) and partial least squares (Ma et al. 2011a). Specifically, Ma et al. (2011a) employ a weighted coexpression network to understand gene interactions and use PCA to reduce gene expression dimensionality. They investigate higher-order representative features, including principal components beyond the first- and second-order terms, with two gradient thresholding methods for feature selection and regularized estimation.

### 4.2. Nonlinear Effects–Based Analysis

There are also approaches based on the more recent deep neural network techniques for accommodating nonlinear effects. For instance, the neural networks proposed by Günther et al. (2012) offer the advantage of avoiding a prior transformation of variables and implicitly modeling interactions without requiring a prior formulation. To assess modeling capability, Günther et al. (2012) define theoretical risk models representing various two-way interactions and conduct evaluation by comparing predicted risk with theoretical risk. Recent examples also include the framework proposed by Zhang et al. (2022), called Deep-DPGI, for detecting high-order gene interactions, utilizing a combination of deep learning and differential privacy. Deep-DPGI disrupts neuron weights through an adaptive noising mechanism, ensuring the privacy of high-order gene interactions while balancing privacy and utility.

Recently, much effort has been devoted to improving the interpretability of prediction-based analysis, where sparse techniques have been commonly adopted. For example, Wu et al. (2023b) develop a deep neural network designed to handle censored survival data, combined with penalization techniques. This method is capable of conducting model estimation and selection simultaneously. Notably, it uniquely preserves the main effects–interactions hierarchy, thereby ensuring that the analysis results offer interpretability comparable to that of traditional regression-based analysis.

## 5. STATISTICAL PROPERTIES

Establishing statistical properties for high-dimensional analysis is inherently challenging. For G–E interaction analysis, this is further complicated by the hierarchical structure, coefficient decomposition, and so on. As a result, the development of statistical properties in G–E interaction research has been somewhat limited. Here, we demonstrate the establishment of statistical properties through two examples of joint analysis: the first utilizes a testing-based approach, and the second is grounded in an estimation-based approach.

Consider the testing-based approach of Liang et al. (2024), where the asymptotic normality of the debiased lasso estimator is established. Furthermore, they demonstrate that the proposed procedure controls the asymptotic FDR hierarchically under high-dimensional settings. Denote as $\xi_{0}$ the d-dimensional coefficient vector consisting of all true regression coefficients. Let $\hat{\xi }$ represent the lasso penalized estimator, and ${\hat{\xi }}^{d}$ represent the debiased lasso estimator, which is formulated as

15. $${\hat{\xi }}^{d}=\hat{\xi }+\frac{1}{n}\hat{M}\Phi^{⊤}H\left(y-\Phi \hat{\xi }\right).$$

Here, $\hat{M}$ is the d×d decorrelating matrix, and Φ represents the augmented design matrix that contains genetic factors, environmental factors, and G–E interactions. H is the n×n rescaled weight matrix, and y is the minimum of the logarithms of the event and censoring times. Based on Equation 15, Liang et al. (2024) derive the following expression to facilitate the establishment of asymptotic normality:

16. $$\sqrt{n}\left({\hat{\xi }}^{d}-\xi_{0}\right)=\frac{1}{\sqrt{n}}\hat{M}\Phi^{⊤}H\varepsilon -\sqrt{n}\left(\hat{M}\hat{\Gamma }-I_{d}\right)\left(\hat{\xi }-\xi_{0}\right),$$

where $\hat{\Gamma }=\Phi^{⊤}H\Phi /n$ is the empirical weighted covariance matrix, and $I_{d}$ is an d×d identity matrix.

Denote the truly important effect index set $A=\left\{j\in \left[d\right]:\xi_{0,j}\neq 0\right\}$, $v=\hat{M}\Phi^{⊤}H\varepsilon \text{/}\sqrt{n}$, and $\Delta =\sqrt{n}\left(\hat{M}\hat{\Gamma }-I_{d}\right)\left(\hat{\xi }-\xi_{0}\right)$. $\hat{\Sigma }$ is the sample covariance matrix associated with the observed data. Define $\hat{\Lambda }=\hat{M}\hat{\Sigma }{\hat{M}}^{⊤}$, and Λ represents its population counterpart. To establish statistical properties, certain conditions need to be satisfied. For example, the censoring indicator and the covariance matrix Φ are required to be conditionally independent given the failure time. Furthermore, the error term ε follows a sub-Gaussian distribution. Under these and some other conditions, when $\left|A^{c}\right|\geq cd$ for a constant 00<c≤1, $|A|\sqrt{\log\;d\text{/}n}=o\left(1\right)$, the tuning parameter $\lambda =O\left(\sqrt{\log\;d\text{/}n}\right)$, and $\mu =O\left(\sqrt{\log\;d\text{/}n}\right)$, we have

1. $v_{j}\overset{d\text{​}}{\to }N\left(0,{\text{Λ}}_{jj}\right)$, where $v_{j}=\sqrt{n}\sum_{i=1}^{n}b_{i}{\hat{m}}_{j}^{⊤}\phi_{i}\varepsilon_{i}$.
2. If, additionally, $\sqrt{n}\lambda \mu |A|\to 0$, then $\|\Delta {\|}_{\infty }=o_{p}\left(1\right)$, where $\Delta =\sqrt{n}\left(\hat{M}\hat{\Gamma }-I_{d}\right)\left(\hat{\xi }-\xi_{0}\right)$.

Here, ${\text{Λ}}_{jj}$represents the jth diagonal entry of Λ, λ controls the sparsity of the lasso, and μ controls the entry-wise $l_{\infty }$ norm of $\hat{M}\hat{\Gamma }-I_{d}$ and the bias of ${\hat{\xi }}^{d}$. In this theorem, the entrywise asymptotic normality of v is established in result 1, and result 2 establishes that, compared with v, the noise term Δ is asymptotic negligible.

Define the normalized matrix $\Lambda^{0}$ as ${\text{Λ}}_{jk}^{0}={\text{Λ}}_{jk}\text{/}\sqrt{{\text{Λ}}_{jj}{\text{Λ}}_{kk}}$. For a given constant q>0, $Q\left(q,b\right)\equiv \left\{\left(i,j\right):1\leq i,j\leq d,\left|{\text{Λ}}_{ij}^{0}\right|\geq b{\left(\log\;d\right)}^{-2-q}\right\}$ for a certain positive constant b. Under the aforementioned conditions, given positive constants b and q, suppose that $|Q\left(q,b\right)|=o\left(d^{1+\rho }\right)$ for some ρ∈[0,1), and $\left|\left\{\left(i,j\right):\left|{\text{Λ}}_{ij}^{0}\right|>\left(1-\rho \right)\text{/}\left(1+\rho \right)\right\}\right|=O\left(d\right)$, and then, for the hierarchical FDR control procedure,

17. $$\underset{\left(n,p\right)\to \infty }{\text{lim}\;\text{sup}}\text{FDR}\leq \alpha .$$

Here, *α* is the prespecified level.

Estimation-based G–E interaction analysis approaches differ from the previously discussed testing-based interaction analysis, as they usually put more emphasis on the estimation and selection consistency of the estimators. For example, Wu et al. (2020) develop a structured G–E interaction estimator and establish its estimation and selection consistency under high-dimensional settings with $\log\left(p\right)=O\left(n^{a}\right)$, $a\in \left(0,\frac{1}{2}\right)$. Specifically, consider the model in Equation 12, with $g\left(\mu_{i}\right)=\mu_{i}$ for continuous $y_{i}$ and $\epsilon_{i}$ being the random error. Denote as ξ the unknown p+K+pK–dimensional coefficient vector consisting of all main environmental effects, main genetic effects, and interactions; $\xi_{0}$ as the corresponding true parameter values; and A as the index set of the nonzero elements of $\xi_{0}$. Wu et al. (2020) first consider the oracle estimator $\xi_{A}^{*}$, where the true sparsity structure is known. Under some conditions (including that the residuals are independently and identically distributed following a sub-Gaussian distribution; the size of the smallest signal is limited by a lower bound; the nonzero effects vanish asymptotically at a rate not exceeding $\sqrt{s\text{/}n}$; both the predictor matrix and J are well-behaved; and the tuning parameters satisfy the orders related with n and p, where s=|A| is the size of the true sparsity and the matrix J of dimension p×p accommodates the structural aspects of the G measurements), it is proved that

18. $$∥\xi_{A}^{*}-\xi_{0,A}{∥}_{2}=O_{p}\left(\sqrt{s\text{/}n}\right).$$

That is, the oracle estimator has the estimation consistency.

Based on Equation 18, Wu et al. (2020) further define an estimator $\hat{\xi }$ with ${\hat{\xi }}_{A}=\xi_{A}^{*}$ and ${\hat{\xi }}_{A_{1}^{c}}=0$ and show that with probability tending to 1, $\hat{\xi }$ is a strict local minimizer of the proposed objective function. This result demonstrates that the proposed estimator performs as well as the oracle one ${\hat{\xi }}_{A}^{*}$ and thus enjoys both the selection and estimation consistency properties.

## 6. COMPUTATIONAL DEVELOPMENTS

In testing-based analysis, the algorithms are relatively simple, because usually only the statistics need to be calculated. For estimation-based analysis, the algorithms for marginal analysis are generally not complicated, except for some special cases, such as the rank-based estimation approach of Shi et al. (2014), whose objective function (Equation 9) is nonconvex. In this case, direct maximization is non-deterministic polynomial-time hard (NP-hard). A smooth approximation and coordinate descent are used to optimize this objective function. The joint analysis methods encounter a substantial increase in computational complexity due to the need to handle a large number of factors simultaneously. Algorithms such as coordinate descent and alternating direction method of multipliers (ADMM) are widely used for optimization. Many models involve tuning parameters, which are typically used for controlling model sparsity, especially in the estimation-based methods. To choose tuning parameters, approaches such as the Bayesian information criterion and cross-validation are commonly used. In Bayesian analysis, MCMC methods such as the Gibbs sampler and the Metropolis–Hastings algorithm are widely utilized for sampling.

Some studies have made their computer programs and packages available to the public. They often are tailored to specific data settings and analysis methods. For example, the CQPCorr method proposed by Xu et al. (2019) addresses overall survival, while the StructLMM method proposed by Moore et al. (2019) deals with continuous phenotypes. In Table 1, we present a list of software for some of the methods discussed, including their specific types or programming languages, along with the URLs where they can be accessed and the outcome types they address. A variety of software environments and programming languages have been used, from Java and Matlab to a collection of R packages. Notably, R codes and packages predominate as the most common type of software, usually available on GitHub and CRAN. In addition, some developers host their programs on personal or laboratory websites. For example, the LEMMA software, adept at dissecting G–E interactions and estimating environmental scores for use in large datasets like the UK Biobank, is a C++-based tool available on the lead developer’s personal site. Similarly, GMDR, which is Java-based software, is hosted on the lab website of Zhejiang University’s Institute of Bioinformatics.

## 7. APPLICATION

### 7.1. Data Preprocessing

Before conducting data analysis, preprocessing is often needed. In practical applications, differences in data scale between environmental and genetic variables are common, along with variations in data types. To ensure uniformity across diverse variables, standardization can be conducted as a preliminary step. In addition, the management of missing data remains a critical consideration. While the issue of missing genetic measurements is becoming less prevalent due to technological advancements, missing environmental measurements persist as an inevitable challenge, particularly in the realm of epidemiological research. Failing to adequately address missingness can result in biased estimates and erroneous identification of significant markers (Wu et al. 2017). Moreover, prescreening is sometimes necessary. The dimensionality of some genetic measurements can be drastically high. From a computational perspective, as software and hardware capabilities improve, analysis can accommodate increasingly high-dimensional data. From a theoretical perspective, some recent studies have established consistency properties under ultra high-dimensional settings, where the dimension *p* can grow exponentially fast with the sample size. However, sample sizes remain relatively small in practical applications, making prescreening often necessary as a way of improving estimation quality, such as improving stability and controlling computational cost. Common methods for prescreening include approaches based on variance, minor allele frequency (MAF), and biological pathways. For example, Lu et al. (2021) perform prescreening by removing SNPs with MAF *<* 0.05, ensuring that only genetic variants with a higher frequency in the population are included in analysis.

### 7.2. Overview of Applications

The aforementioned methodologies, along with other G–E interaction approaches, have been extensively applied to a wide range of complex diseases, yielding significant biological insights and meaningful discoveries. A selection of applications using real data is summarized in Table 2. These methods have been applied to investigate various diseases, such as lung cancer, nonalcoholic fatty liver disease, breast cancer, pancreatic cancer, and depression. The outcomes range from binary disease status to overall survival. The environmental factors are also diverse, including lifestyle habits such as smoking, biomarkers like body mass index and cholesterol levels, sociodemographic variables such as gender, and clinical treatments. The genetic factors are typically SNPs and gene expressions. Each method is also followed by a reference to a specific section that provides a more detailed description.

In the application of various methods to real data, differences in sample size and dimensionality can be significant factors contributing to the difference in the number of identified interactions. For example, de Rochemonteix et al. (2021), when demonstrating the proposed method on lung cancer, use data from a National Cancer Institute genome-wide association study that involves 5,739 cases and 5,848 controls. The investigators use 14 SNPs as genetic factors and smoking, a binary variable, as the environmental factor. Three interactions are identified. Wu & Ma (2019b), in an application to stomach adenocarcinoma, use data from The Cancer Genome Atlas that include 386 samples. After prescreening, 2,000 gene expressions from the original 20,189 gene expression measurements are used for downstream analysis. The environmental factors include age; American Joint Committee on Cancer (AJCC) metastasis pathologic stage; AJCC nodes pathologic stage; AJCC tumor pathologic stage; gender; *International Classification of Diseases for Oncology*, third edition (ICD O3) histology; ICD O3 site; and history of other malignancy. Three environmental effects (age, AJCC metastasis pathologic stage, and gender), 45 genetic effects, and 23 G–E interactions are identified.

## 8. CONCLUSIONS

In this review, we have presented various approaches for analyzing the interactions between low-dimensional environmental factors and high-dimensional genetic factors. With particular attention to the high dimensionality of the genetic factors, we have concentrated on three main families of statistical techniques: hypothesis testing, variable selection, and dimension reduction. Different assumptions and goals have been considered in different approaches, leading to different application scenarios. We have also discussed the statistical properties, computations, and applications of some representative approaches.

Compared with only considering main effects, there are generally more challenges in G–E interaction analysis. For testing-based analysis, the approaches for these two situations are often similar. However, with the inclusion of G–E interactions, the number of multiple comparisons increases, making the testing more stringent. In addition, nonlinear effects are often considered in interaction analysis, which brings more challenges than only considering main effects. The main effects–interactions hierarchical structure is sometimes considered in joint analysis, adding further complexity. For example, the hierarchical structure needs to be accounted for when calculating statistics and *p*-values in testing-based analysis. Additionally, with estimation-based methods, the consideration of hierarchical structure can make penalty terms more complex, and some methods may involve decomposing coefficients, which significantly increases both theoretical and computational complexity. Moreover, in Bayesian analysis, priors with sparsity, such as spike-and-slab priors, may be necessary if the dimension is high, and the priors must be set more thoughtfully when the hierarchical structure is considered.

Despite rapid developments and exciting successes, there are still many open questions that demand further research. For example, the majority of studies to date have concentrated on continuously distributed gene expressions and SNPs with moderate to high MAFs. Rare features with weak signals, such as SNPs with low MAFs, have been less examined. The environmental factors have usually been preselected in the existing studies and not subject to selection. However, in some environment-wide association studies, a large number of environmental factors have been collected, demanding further selection and regularization in interaction models. Advanced supervised artificial intelligence techniques, such as deep learning, transfer learning, and active learning, also bring new directions for G–E interaction analysis. Due to their black-box properties, many efforts are needed to enhance interpretability. In addition, compared with methodology developments, investigations on statistical properties and development of user-friendly software are still limited. It is expected that G–E interaction analysis will continue to be an intense subject of statistical and biomedical research, being an important tool for comprehending the intricate molecular mechanisms of complex diseases.

## Figures and Tables

**Table 1:** Summary of software (partial list)

Approach	Software type	Website	Outcome type
aGE (Yang et al. 2019)	R package	http://github.com/ytzhong/projects/	Binary
BQRVCSS (Zhou et al. 2023a)	R package	https://github.com/cenwu/pqrBayes	Continuous
BSSVC-SI (Ren et al. 2020)	R package	https://cran.r-project.org/web/packages/spinBayes/index.html	Continuous
CQPCorr (Xu et al. 2019)	R code	https://github.com/shuanggema/CQPCorr	Survival
fastGWA-GE (Zhong et al. 2023)	C++ code	https://github.com/jianyangqt/gcta.git	Continuous
FastKM (Marceau et al. 2015)	R package	https://cran.r-project.org/web/packages/FastKM/index.html	Binary, continuous, survival
GEM (Westerman et al. 2021)	C++ code	https://github.com/large-scale-gxe-methods/GEM	Binary continuous
gesso (Zemlianskaia et al. 2022)	R package	https://CRAN.R-project.org/package=gesso	Continuous
GMDR (Hou et al. 2019)	Java code	http://ibi.zju.edu.cn/software	Ordinal
IPCW-pcorr (Wang & Yang 2022)	R code	https://doi.org/10.6084/m9.figshare.19306967.v3	Survival
LAD-hier (Wu et al. 2018)	R code	https://github.com/cenwu/RobustHierGXE	Survival
LEMMA (Kerin & Marchini 2020)	C++ code	https://github.com/mkerin/LEMMA	Continuous
LRT-R (de Rochemonteix et al. 2021)	R package	https://www.bioconductor.org/packages/release/bioc/html/CGEN.html	Binary
MixGE (Wang et al. 2017)	Matlab code	https://github.com/bieqa/MixGE	Binary continuous
psgMCP (Wang et al. 2019)	R code	https://github.com/Xu-Yonghong/psgMCP	Continuous
RBSG-SS (Ren et al. 2023)	R package	https://cran.r-project.org/web/packages/roben/index.html	Continuous
RITSS (Hecker et al. 2022)	R package	https://github.com/julianhecker/RITSS	Continuous
sail (Bhatnagar et al. 2023)	R package	https://github.com/sahirbhatnagar/sail	Binary continuous
StructLMM (Moore et al. 2019)	Python code	https://github.com/limix/struct-lmm	Continuous

Abbreviations: aGE, adaptive gene–environment interaction; BQRVCSS, Bayesian regularized quantile varying coefficient model with spike-and-slab priors; BSSVC-SI, Bayesian spike-and-slab variable selection with structural identification; CQPCorr, censored quantile partial correlation; GEM, gene–environment interaction analysis for millions of samples; GMDR, proportional odds model–based multifactor dimensionality reduction for ordinal phenotypes; IPCW-pcorr, inverse probability-of-censoring weighted Kendall’s partial correlation approach; LAD, least absolute deviation; LEMMA, linear environment mixed model analysis; LRT-R, retrospective likelihood ratio testing; MixGE, mixed effect model for gene–environment interaction; psgMCP, prior sparse group minimum concave penalty; RBSG-SS, robust Bayesian sparse group selection with spike-and-slab priors; RITSS, robust interaction testing using sample splitting; StructLMM, structured linear mixed model.

**Table 2:** Summary of applications (partial list)

Reference	Method	Disease	Outcome	Interaction
Chai et al. (2017)	This method employs an AFT model to characterize prognosis, incorporating the exponential squared loss to accommodate data contamination or a mixture of outcome distributions. Concurrently, it utilizes a penalization strategy for regularized estimation and variable selection (Section 3.1.1).	Lung squamous cell carcinoma	Overall survival	E: age, gender, smoking pack years, smoking status G: gene expression
de Rochemonteix et al. (2021)	LRT-R identifies additive G–E interactions by leveraging the trend effect of a genotype and further harnessing the independence between genes and the environmental factors for enhanced detection (Section 2.1.1).	Lung cancer	Binary case-control	E: smoking G: SNP
		Late-onset Alzheimer’s disease	Binary case-control	E: gender, APOE *ε*4 G: SNP
Liang et al. (2024)	This study introduces a hierarchical FDR control approach for high-dimensional survival analysis with interactions, utilizing the AFT model for survival and a weighted least squares plus debiased lasso technique for estimation and selection (Section 2.2.1).	Breast cancer	Overall survival	E: nonsynonymous tumor mutation burden, age at diagnosis, estrogen receptor status G: gene expression
Lu et al. (2021)	LADBLSS is a robust Bayesian method, incorporating the spike-and-slab priors and implementing the Gibbs sampling based on MCMC (Section 3.1.2).	Type 2 diabetes	Weight	E: age, total physical activity, trans fat intake, cereal fiber intake, reported high blood cholesterol G: SNP
Pashova et al. (2017)	Directed lasso is a regression modeling strategy designed for identifying interactions between genes and treatments or environmental factors by using a structured interaction model and a pairwise fused lasso penalty (Section 3.2.1).	Breast cancer	Overall survival	E: treatment G: gene expression
Ren et al. (2023)	This fully Bayesian robust variable selection method effectively handles heavy-tailed errors and outliers in the response variable while conducting variable selection through structural sparsity. By applying the spike-and-slab priors at both the individual and group levels, it robustly identifies significant main effects and interactions (Section 3.2.2).	Type 2 diabetes	Weight	E: total physical activity, glycemic load, cereal fiber intake, alcohol intake, history of high cholesterol G: SNP
		Cutaneous melanoma	Breslow’s depth	E: age, AJCC tumor pathologic stage, gender, Clark level G: gene expression
Wang et al. (2019)	psgMCP is a quasi-likelihood-based approach for identifying G–E interactions and main genetic effects, integrating information from the existing literature, and employing a penalization method for identification and selection that respects the main effects–interactions hierarchy (Section 3.2.1).	Cutaneous melanoma	Overall survival	E: age, AJCC tumor pathologic stage, gender, Clark level at diagnosis G: gene expression
		Glioblastoma multiforme	Overall survival	E: age, gender, Karnofsky performance score, race G: gene expression
Wu & Ma (2019b)	This method combines a semiparametric model with the Huber loss function and *Q*_*n*_ estimator for robust analysis of G–E interactions, accommodating nonlinear effects and data contamination. It utilizes sparse boosting for selection and regression-based imputation for missing data, while respecting the main effects–interactions hierarchy (Section 3.2.1).	Stomach adenocarcinoma	Overall survival	E: age, AJCC metastasis pathologic stage, AJCC nodes pathologic stage, AJCC tumor pathologic stage, gender, ICD O3 histology, ICD O3 site, history of other malignancy G: gene expression
		Cutaneous melanoma	Breslow’s depth	E: weight, height, Clark level, age, AJCC metastasis pathologic stage, AJCC nodes pathologic stage, AJCC tumor pathologic stage, gender, and sample type G: gene expression
Wu et al. (2023b)	This method combines deep neural networks with penalization techniques. It conducts model estimation and selection, respects the hierarchical structure of main effects–interactions in variable selection, and leverages the strengths of neural networks and regression analysis for enhanced interpretability and effectiveness (Section 4.2).	Lung adenocarcinoma	Overall survival	E: age, gender, AJCC tumor pathologic stage, Clark level, Breslow’s depth at diagnosis G: gene expression
Yang et al. (2019)	Designed under the aSPU framework, the aGE test incorporates spline functions to address potential type I error inflation from model misspecification, aiming to efficiently detect genetic main effects among numerous neutral rare variants (Section 2.2.1).	Pancreatic cancer	Binary case-control	E: smoking pack years G: rare variant
Zhao et al. (2019)	The composite kernel approach models the overall genetic effect of a SNP set by incorporating potential G–E interactions through a weighted average of two separate kernels representing the genetic main effects and the G–E interaction effects, respectively. The weights are estimated in a data-dependent manner to enhance statistical power of the proposed restricted likelihood ratio test (Section 2.2.2).	Depression	Beck Depression Inventory	E: gender G: SNP
Zhou et al. (2023b)	The semiparametric VC model identifies G–E interactions for continuous traits, capturing both linear and nonlinear trajectories without a predefined model. By utilizing a spline function within an LMM framework, it dynamically models genetic and G–E effects, enhancing detection capabilities (Section 2.1.2).	Nonalcoholic fatty liver disease	Hepatic triglyceride content	E: BMI G: SNP

Abbreviations: AFT, accelerated failure time; aGE, adaptive gene–environment interaction; AJCC, American Joint Committee on Cancer; APOE, apolipoprotein E; aSPU, adaptive sum of powered score; BMI, body mass index; E, environmental; FDR, false discovery rate; G, genetic; G–E, gene–environment; ICD O3, *International Classification of Diseases for Oncology*, third edition; LADBLSS, least absolute deviation Bayesian lasso with spike-and-slab priors; LMM, linear mixed model; LRT-R, retrospective likelihood ratio testing; MCMC, Markov chain Monte Carlo; psgMCP, prior sparse group minimum concave penalty; SNP, single nucleotide polymorphism; VC, varying coefficient.

## References

[R1] Alonso-Curbelo D, Ho YJ, Burdziak C, Maag JL, Morris JP IV, 2021. A gene–environment-induced epigenetic program initiates tumorigenesis. Nature 590(7847):642–48 33536616 10.1038/s41586-020-03147-x PMC8482641

[R2] Berghöfer B, Frommer T, Haley G, Fink L, Bein G, Hackstein H. 2006. TLR7 ligands induce higher IFN-*α* production in females. J. Immunol 177(4):2088–96 16887967 10.4049/jimmunol.177.4.2088

[R3] Bhatnagar SR, Lu T, Lovato A, Olds DL, Kobor MS, 2023. A sparse additive model for high-dimensional interactions with an exposure variable. Comput. Stat. Data Anal 179:107624

[R4] Chai H, Zhang Q, Jiang Y, Wang G, Zhang S, 2017. Identifying gene-environment interactions for prognosis using a robust approach. Econom. Stat 4:105–20 31157309 10.1016/j.ecosta.2016.10.004 PMC6541416

[R5] Cheng RYS, Hockman T, Crawford E, Anderson LM, Shiao YH. 2004. Epigenetic and gene expression changes related to transgenerational carcinogenesis. Mol. Carcinog 40(1):1–11 15108325 10.1002/mc.20022

[R6] Chiu CY, Wang S, Zhang B, Luo Y, Simpson C, 2022. Gene-level association analysis of ordinal traits with functional ordinal logistic regressions. Genet. Epidemiol 46(5–6):234–55 35438198 10.1002/gepi.22451 PMC11550389

[R7] Dai JY, Kooperberg C, Leblanc M, Prentice RL. 2012. Two-stage testing procedures with independent filtering for genome-wide gene-environment interaction. Biometrika 99(4):929–44 23843674 10.1093/biomet/ass044 PMC3629859

[R8] de Rochemonteix M, Napolioni V, Sanyal N, Belloy ME, Caporaso NE, 2021. A likelihood ratio test for gene-environment interaction based on the trend effect of genotype under an additive risk model using the gene-environment independence assumption. Am. J. Epidemiol 190(1):129–41 32870973 10.1093/aje/kwaa132 PMC8979298

[R9] Fan R, Wang Y, Mills JL, Wilson AF, Bailey-Wilson JE, Xiong M. 2013. Functional linear models for association analysis of quantitative traits. Genet. Epidemiol 37(7):726–42 24130119 10.1002/gepi.21757 PMC4163942

[R10] Fan R, Zhong M, Wang S, Zhang Y, Andrew A, 2011. Entropy-based information gain approaches to detect and to characterize gene-gene and gene-environment interactions/correlations of complex diseases. Genet. Epidemiol 35(7):706–21 22009792 10.1002/gepi.20621 PMC3384547

[R11] Fang K, Li J, Zhang Q, Xu Y, Ma S. 2023. Pathological imaging-assisted cancer gene–environment interaction analysis. Biometrics 79(4):3883–94 37132273 10.1111/biom.13873 PMC10622332

[R12] Günther F, Pigeot I, Bammann K. 2012. Artificial neural networks modeling gene-environment interaction. BMC Genet 13:37 22583704 10.1186/1471-2156-13-37 PMC3507700

[R13] Hahn LW, Ritchie MD, Moore JH. 2003. Multifactor dimensionality reduction software for detecting gene–gene and gene–environment interactions. Bioinformatics 19(3):376–82 12584123 10.1093/bioinformatics/btf869

[R14] Han SS, Chatterjee N. 2018. Review of statistical methods for gene-environment interaction analysis. Curr. Epidemiol. Rep 5:39–45

[R15] Han SS, Rosenberg PS, Garcia-Closas M, Figueroa JD, Silverman D, 2012. Likelihood ratio test for detecting gene (G)-environment (E) interactions under an additive risk model exploiting G-E independence for case-control data. Am. J. Epidemiol 176(11):1060–67 23118105 10.1093/aje/kws166 PMC3571244

[R16] Hecker J, Prokopenko D, Moll M, Lee S, Kim W, 2022. A robust and adaptive framework for interaction testing in quantitative traits between multiple genetic loci and exposure variables. PLOS Genet 18(11):e1010464 36383614 10.1371/journal.pgen.1010464 PMC9668174

[R17] Hou TT, Lin F, Bai S, Cleves MA, Xu HM, Lou XY. 2019. Generalized multifactor dimensionality reduction approaches to identification of genetic interactions underlying ordinal traits. Genet. Epidemiol 43(1):24–36 30387901 10.1002/gepi.22169 PMC8495755

[R18] Jiang Y, Chiu CY, Yan Q, Chen W, Gorin MB, 2021. Gene-based association testing of dichotomous traits with generalized functional linear mixed models using extended pedigrees: applications to age-related macular degeneration. J. Am. Stat. Assoc 116(534):531–45 34321704 10.1080/01621459.2020.1799809 PMC8315575

[R19] Kawaguchi ES, Kim AE, Lewinger JP, Gauderman WJ. 2023. Improved two-step testing of genome-wide gene–environment interactions. Genet. Epidemiol 47(2):152–66 36571162 10.1002/gepi.22509 PMC9974838

[R20] Kerin M, Marchini J. 2020. Inferring gene-by-environment interactions with a Bayesian whole-genome regression model. Am. J. Hum. Genet 107(4):698–713 32888427 10.1016/j.ajhg.2020.08.009 PMC7536582

[R21] Kim J, Ziyatdinov A, Laville V, Hu FB, Rimm E, 2019. Joint analysis of multiple interaction parameters in genetic association studies. Genetics 211(2):483–94 30578273 10.1534/genetics.118.301394 PMC6366922

[R22] Knights J, Yang J, Chanda P, Zhang A, Ramanathan M. 2013. Symphony, an information-theoretic method for gene–gene and gene–environment interaction analysis of disease syndromes. Heredity 110(6):548–59 23423149 10.1038/hdy.2012.123 PMC3656633

[R23] Kraft P, Yen YC, Stram DO, Morrison J, Gauderman WJ. 2007. Exploiting gene-environment interaction to detect genetic associations. Hum. Hered 63(2):111–19 17283440 10.1159/000099183

[R24] Li Y, Wang F, Wu M, Ma S. 2022. Integrative functional linear model for genome-wide association studies with multiple traits. Biostatistics 23(2):574–90 33040145 10.1093/biostatistics/kxaa043 PMC9007435

[R25] Liang W, Zhang Q, Ma S. 2024. Hierarchical false discovery rate control for high-dimensional survival analysis with interactions. Comput. Stat. Data Anal 192:107906 38098875 10.1016/j.csda.2023.107906 PMC10718515

[R26] Lin X, Lee S, Christiani DC, Lin X. 2013. Test for interactions between a genetic marker set and environment in generalized linear models. Biostatistics 14(4):667–81 23462021 10.1093/biostatistics/kxt006 PMC3769996

[R27] Liu C, Ma J, Amos CI. 2015. Bayesian variable selection for hierarchical gene–environment and gene–gene interactions. Hum. Genet 134:23–36 25154630 10.1007/s00439-014-1478-5 PMC4282989

[R28] Liu J, Huang J, Zhang Y, Lan Q, Rothman N, 2013. Identification of gene–environment interactions in cancer studies using penalization. Genomics 102(4):189–94 23994599 10.1016/j.ygeno.2013.08.006 PMC3869641

[R29] Liu X, Zhong PS, Cui Y. 2020. Joint test of parametric and nonparametric effects in partial linear models for gene-environment interaction. Stat. Sin 30(1):325–46

[R30] Lobach I, Mallick B, Carroll RJ. 2011. Semiparametric Bayesian analysis of gene-environment interactions with error in measurement of environmental covariates and missing genetic data. Stat. Interface 4(3):305–15 21949562 10.4310/sii.2011.v4.n3.a5 PMC3178196

[R31] Lou XY, Chen GB, Yan L, Ma JZ, Zhu J, 2007. A generalized combinatorial approach for detecting gene-by-gene and gene-by-environment interactions with application to nicotine dependence. Am. J. Hum. Genet 80(6):1125–37 17503330 10.1086/518312 PMC1867100

[R32] Lu M, Lee HS, Hadley D, Huang JZ, Qian X. 2014. Logistic principal component analysis for rare variants in gene-environment interaction analysis. IEEE/ACM Trans. Comput. Biol. Bioinform 11(6):1020–28 26357039 10.1109/TCBB.2014.2322371

[R33] Lu X, Fan K, Ren J, Wu C. 2021. Identifying gene–environment interactions with robust marginal Bayesian variable selection. Front. Genet 12:667074 34956304 10.3389/fgene.2021.667074 PMC8693717

[R34] Ma S, Kosorok MR, Huang J, Dai Y. 2011a. Incorporating higher-order representative features improves prediction in network-based cancer prognosis analysis. BMC Med. Genom 4:5 10.1186/1755-8794-4-5 PMC3037289 21226928

[R35] Ma S, Yang L, Romero R, Cui Y. 2011b. Varying coefficient model for gene–environment interaction: a nonlinear look. Bioinformatics 27(15):2119–26 21690105 10.1093/bioinformatics/btr318 PMC3137212

[R36] Maity A, Carroll RJ, Mammen E, Chatterjee N. 2009. Testing in semiparametric models with interaction, with applications to gene–environment interactions. J. R. Stat. Soc. Ser. B 71(1):75–96 10.1111/j.1467-9868.2008.00671.x PMC2762226 19838317

[R37] Majumdar A, Burch KS, Haldar T, Sankararaman S, Pasaniuc B, 2020. A two-step approach to testing overall effect of gene–environment interaction for multiple phenotypes. Bioinformatics 36(24):5640–48 10.1093/bioinformatics/btaa1083 33453114

[R38] Manuck SB, McCaffery JM. 2014. Gene-environment interaction. Annu. Rev. Psychol 65:41–70 24405358 10.1146/annurev-psych-010213-115100

[R39] Marceau R, Lu W, Holloway S, Sale MM, Worrall BB, 2015. A fast multiple-kernel method with applications to detect gene-environment interaction. Genet. Epidemiol 39(6):456–68 26139508 10.1002/gepi.21909 PMC4544636

[R40] McAllister K, Mechanic LE, Amos C, Aschard H, Blair IA, 2017. Current challenges and new opportunities for gene-environment interaction studies of complex diseases. Am. J. Epidemiol 186(7):753–61 28978193 10.1093/aje/kwx227 PMC5860428

[R41] Miao J, Wu Y, Lu Q. 2024. Statistical methods for gene–environment interaction analysis. Wiley Interdiscip. Rev. Comput. Stat 16(1):e1635 38699459 10.1002/wics.1635 PMC11064894

[R42] Migliore L, Coppedè F. 2022. Gene–environment interactions in Alzheimer disease: the emerging role of epigenetics. Nat. Rev. Neurol 18(11):643–60 36180553 10.1038/s41582-022-00714-w

[R43] Moore R, Casale FP, Jan Bonder M, Horta D, Franke L, 2019. A linear mixed-model approach to study multivariate gene–environment interactions. Nat. Genet 51(1):180–86 30478441 10.1038/s41588-018-0271-0 PMC6354905

[R44] Pashova H, LeBlanc M, Kooperberg C. 2017. Structured detection of interactions with the directed lasso. Stat. Biosci 9:676–91 29292402 10.1007/s12561-016-9184-6 PMC5747322

[R45] Ren J, Zhou F, Li X, Chen Q, Zhang H, 2020. Semiparametric Bayesian variable selection for gene-environment interactions. Stat. Med 39(5):617–38 31863500 10.1002/sim.8434 PMC7467082

[R46] Ren J, Zhou F, Li X, Ma S, Jiang Y, Wu C. 2023. Robust Bayesian variable selection for gene–environment interactions. Biometrics 79(2):684–94 35394058 10.1111/biom.13670 PMC11086965

[R47] Ren M, Zhang S, Ma S, Zhang Q. 2022. Gene–environment interaction identification via penalized robust divergence. Biom. J 64(3):461–80 34725857 10.1002/bimj.202000157 PMC9386692

[R48] Rudolph A, Chang-Claude J, Schmidt MK. 2016. Gene–environment interaction and risk of breast cancer. Br. J. Cancer 114(2):125–33 26757262 10.1038/bjc.2015.439 PMC4815812

[R49] Sa J, Liu X, He T, Liu G, Cui Y. 2016. A nonlinear model for gene-based gene-environment interaction. Int. J. Mol. Sci 17(6):882 27271617 10.3390/ijms17060882 PMC4926416

[R50] Shi X, Liu J, Huang J, Zhou Y, Xie Y, Ma S. 2014. A penalized robust method for identifying gene–environment interactions. Genet. Epidemiol 38(3):220–30 24616063 10.1002/gepi.21795 PMC4356211

[R51] Thomas D 2010. Gene–environment-wide association studies: emerging approaches. Nat. Rev. Genet 11(4):259–72 20212493 10.1038/nrg2764 PMC2891422

[R52] Wang C, Sun J, Greenwood CM, Qiu A. 2017. A set-based mixed effect model for gene-environment interaction and its application to neuroimaging phenotypes. Front. Neurosci 11:255349 10.3389/fnins.2017.00191 PMC5382297 28428742

[R53] Wang JH, Yang CT. 2022. Identification of gene-environment interactions by non-parametric Kendall’s partial correlation with application to TCGA ultrahigh-dimensional survival genomic data. Front. Biosci 27(8):225 10.31083/j.fbl2708225 36042165

[R54] Wang X, Xu Y, Ma S. 2019. Identifying gene-environment interactions incorporating prior information. Stat. Med 38(9):1620–33 30637789 10.1002/sim.8064 PMC6533537

[R55] Westerman KE, Pham DT, Hong L, Chen Y, Sevilla-González M, 2021. GEM: scalable and flexible gene–environment interaction analysis in millions of samples. Bioinformatics 37(20):3514–20 34695175 10.1093/bioinformatics/btab223 PMC8545347

[R56] Wu C, Cui Y, Ma S. 2014. Integrative analysis of gene–environment interactions under a multi-response partially linear varying coefficient model. Stat. Med 33(28):4988–98 25146388 10.1002/sim.6287 PMC4225006

[R57] Wu C, Jiang Y, Ren J, Cui Y, Ma S. 2018. Dissecting gene-environment interactions: a penalized robust approach accounting for hierarchical structures. Stat. Med 37(3):437–56 29034484 10.1002/sim.7518 PMC5827955

[R58] Wu C, Shi X, Cui Y, Ma S. 2015. A penalized robust semiparametric approach for gene–environment interactions. Stat. Med 34(30):4016–30 26239060 10.1002/sim.6609 PMC4715555

[R59] Wu M, Ma S. 2019a. Robust genetic interaction analysis. Brief. Bioinform 20(2):624–37 29897421 10.1093/bib/bby033 PMC6556899

[R60] Wu M, Ma S. 2019b. Robust semiparametric gene-environment interaction analysis using sparse boosting. Stat. Med 38(23):4625–41 31359454 10.1002/sim.8322 PMC6736719

[R61] Wu M, Wang F, Ge Y, Ma S, Li Y. 2023a. Bi-level structured functional analysis for genome-wide association studies. Biometrics 79(4):3359–73 37098961 10.1111/biom.13871 PMC12225970

[R62] Wu M, Zang Y, Zhang S, Huang J, Ma S. 2017. Accommodating missingness in environmental measurements in gene-environment interaction analysis. Genet. Epidemiol 41(6):523–54 28657194 10.1002/gepi.22055 PMC5561007

[R63] Wu M, Zhang Q, Ma S. 2020. Structured gene-environment interaction analysis. Biometrics 76(1):23–35 31424088 10.1111/biom.13139 PMC7028505

[R64] Wu S, Xu Y, Zhang Q, Ma S. 2023b. Gene–environment interaction analysis via deep learning. Genet. Epidemiol 47(3):261–86 36807383 10.1002/gepi.22518 PMC10244912

[R65] Wu X, Jin L, Xiong M. 2009. Mutual information for testing gene-environment interaction. PLOS ONE 4(2):e4578 19238204 10.1371/journal.pone.0004578 PMC2642626

[R66] Xu Y, Wu M, Ma S. 2022. Multidimensional molecular measurements–environment interaction analysis for disease outcomes. Biometrics 78(4):1542–54 34213006 10.1111/biom.13526 PMC9366385

[R67] Xu Y, Wu M, Ma S, Ejaz Ahmed S. 2018. Robust gene–environment interaction analysis using penalized trimmed regression. J. Stat. Comput. Simul 88(18):3502–28 30718937 10.1080/00949655.2018.1523411 PMC6358205

[R68] Xu Y, Wu M, Zhang Q, Ma S. 2019. Robust identification of gene-environment interactions for prognosis using a quantile partial correlation approach. Genomics 111(5):1115–23 30009922 10.1016/j.ygeno.2018.07.006 PMC6335188

[R69] Yang T, Chen H, Tang H, Li D, Wei P. 2019. A powerful and data-adaptive test for rare-variant–based gene-environment interaction analysis. Stat. Med 38(7):1230–44 30460711 10.1002/sim.8037 PMC6399020

[R70] Zemlianskaia N, Gauderman WJ, Lewinger JP. 2022. A scalable hierarchical lasso for gene–environment interactions. J. Comput. Graph. Stat 31(4):1091–103 36793591 10.1080/10618600.2022.2039161 PMC9928188

[R71] Zhang S, Xue Y, Zhang Q, Ma C, Wu M, Ma S. 2020. Identification of gene–environment interactions with marginal penalization. Genet. Epidemiol 44(2):159–96 31724772 10.1002/gepi.22270 PMC7028443

[R72] Zhang Y, Gao Y, Wang H, Wu H, Xia Y, Wu X. 2022. A secure high-order gene interaction detection algorithm based on deep neural network. IEEE/ACM Trans. Comput. Biol. Bioinform 10.1109/TCBB.2022.3214863 36251904

[R73] Zhao N, Zhang H, Clark JJ, Maity A, Wu MC. 2019. Composite kernel machine regression based on likelihood ratio test for joint testing of genetic and gene–environment interaction effect. Biometrics 75(2):625–37 30430548 10.1111/biom.13003

[R74] Zhong W, Chhibber A, Luo L, Mehrotra DV, Shen J. 2023. A fast and powerful linear mixed model approach for genotype-environment interaction tests in large-scale GWAS. Brief. Bioinform 24(1):bbac547 36545787 10.1093/bib/bbac547

[R75] Zhou F, Ren J, Lu X, Ma S, Wu C. 2021. Gene–environment interaction: a variable selection perspective. In Epistasis: Methods and Protocol, ed. Wong KC, pp. 191–223. New York: Springer 10.1007/978-1-0716-0947-7_13 33733358

[R76] Zhou F, Ren J, Ma S, Wu C. 2023a. The Bayesian regularized quantile varying coefficient model. Comput. Stat. Data Anal 187:107808 38746689 10.1016/j.csda.2023.107808 PMC11090482

[R77] Zhou Z, Ku HC, Manning SE, Zhang M, Xing C. 2023b. A varying coefficient model to jointly test genetic and gene–environment interaction effects. Behav. Genet 53(4):374–82 36622576 10.1007/s10519-022-10131-w PMC10277225

[R78] Zou F, Huang H, Lee S, Hoeschele I. 2010. Nonparametric Bayesian variable selection with applications to multiple quantitative trait loci mapping with epistasis and gene–environment interaction. Genetics 186(1):385–94 20551445 10.1534/genetics.109.113688 PMC2940302

